# Commentary: A Cost-Effective Method to Enhance Adenoviral Transduction of Primary Murine Osteoblasts and Bone Marrow Stromal Cells

**DOI:** 10.3389/fendo.2020.00419

**Published:** 2020-06-25

**Authors:** Aikta Sharma, Alice Goring, Claire E. Clarkin

**Affiliations:** School of Biological Sciences, University of Southampton, Southampton, United Kingdom

**Keywords:** osteoblast, extracellular matrix, poly L lysine, Raman spectroscopy, VEGF

## Introduction

Manipulation of gene expression in bone and related-cell types has, to date, contributed substantially to our knowledge surrounding the genetic regulators of bone-cell function, development and importantly, provided clues to the origins of local and systemic bone pathologies (1). However, effective genetic alterations are often limited by the efficiency of the transduction vector, which in bone are further restricted by the limited proliferative potential of osteoblasts (OBs). Addressing this, a 2016 study published in *Bone Research*, by Buo et al. reported that poly-L-lysine (PLL) coatings provide a cost-effective means to improve adenovirus-mediated genetic knockdown in OBs and bone marrow stromal cells (2). However, our recently published findings using Raman spectroscopy showed that PLL coatings alter OB matrix production, restricting their differentiation capacity and ability to undertake matrix mineralization (3). As successful OB maturation is crucial in the process of osteogenesis, we highlight the immediate need to fully characterize the effect of PLL or similar coatings on both bone and related-cell function, and maturation ahead of genetic manipulation with adenoviral vectors.

## Discussion

Buo et al. (2) has demonstrated how PLL could be exploited for adenovirus-Cre-mediated gene knockdown of floxed primary murine OBs using low multiplicity infection. Results showed that the presence of PLL substantially increased the number and intensity of GFP+ cells, transduced with adenovirus-GFP, with a 2 to 5-fold increase in transduction efficiency observed in OBs and bone marrow stromal cells *in vitro* with further improvements reported *in situ*. Furthermore, the group reported that both OB viability and basal mRNA levels of *Runx2, Osx, Bglap*, and *Gja1/Cx43*, markers of osteogenesis, were not significantly altered as a result of the viral transduction or PLL. Whilst this study has provided interesting observations, the effect of PLL on OB function was limited and effects on OB maturation, extracellular matrix (ECM) and mineral deposition was not assessed.

**Figure 1:**
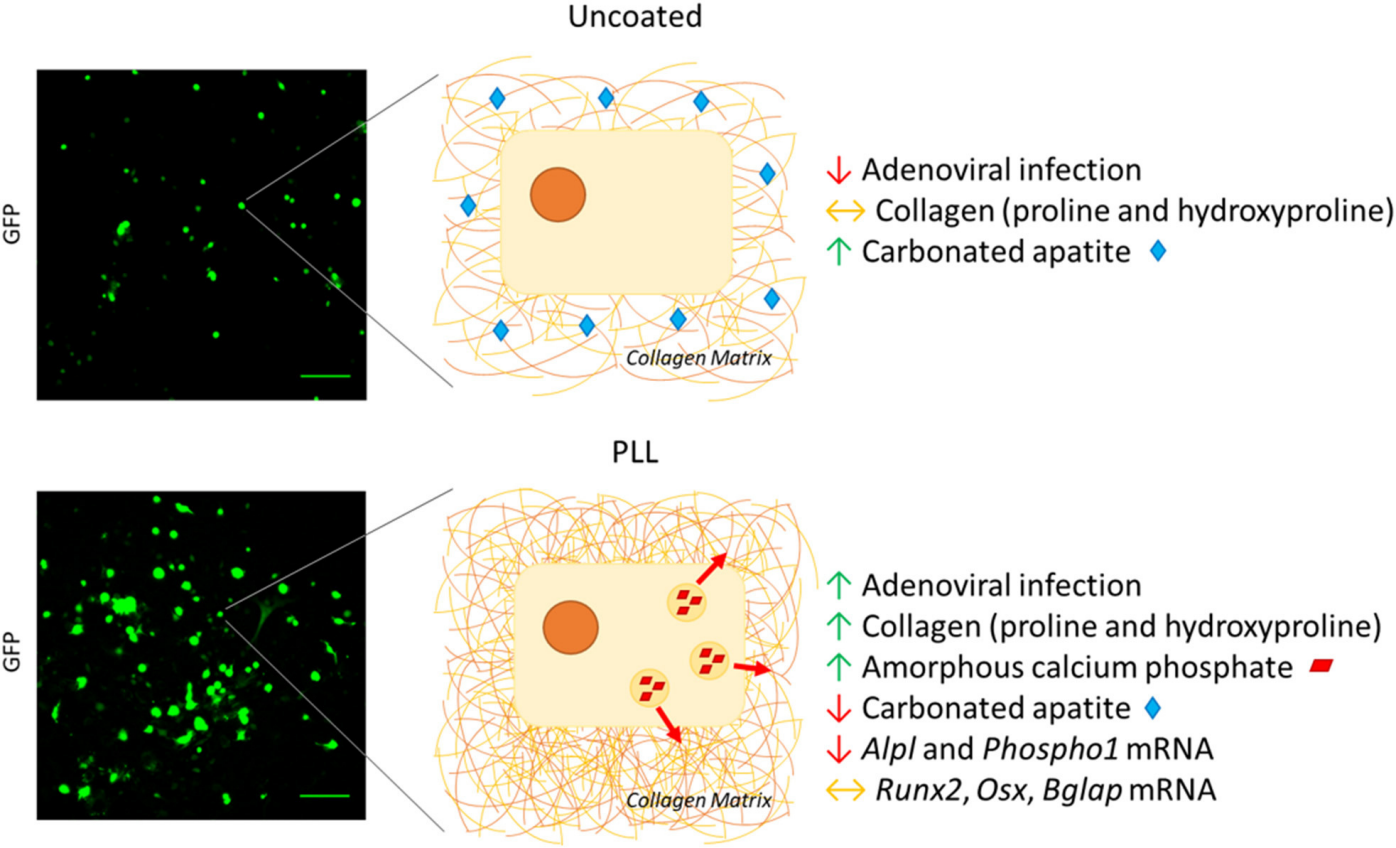
PLL coatings restrict OB maturation. Adenoviral infection visualized by the increased fluorescent GFP-labeling of OBs is improved upon culture on PLL coatings. This improvement in transduction efficiency on PLL is associated with increased presence of collagen-specific proline and hydroxyproline and immature mineral in the form of amorphous calcium phosphate (3). These phases are associated with OB proliferation, with the presence of PLL restricting further OB differentiation that is necessary for matrix mineralization. Here, PLL restricts the formation of carbonated apatite (CAP), the last precursor to hydroxyapatite and is associated with decreased levels of *Alpl* and *Phospho1* transcripts however the expression of early (*Runx2* or *Osx*) or late (*Bglap)* markers of osteogenesis (2) remain unaffected. In the absence of PLL, whilst the adenoviral transduction efficiency is reduced, OBs can progress to the later stages of differentiation whereby CAP is released to mineralize the pre-established collagenous matrix. Scale bars represent 250 μm.

In our previous work, we have highlighted the potential of Raman spectroscopy to sensitively report early proliferation and differentiation-related biochemical changes in primary OB cultures (4). In addition to the favorable qualities of this modality, including its non-invasive and label-free nature, spectral markers of osteogenic differentiation were detected sooner than the methods typically used to assess OB maturation including qPCR, alkaline phosphatase enzymatic activity, and collagen staining. We observed a positive effect of PLL on primary murine long bone-derived OB viability (3). Then using Raman spectroscopy, we examined the composition of OB-derived ECM in more detail, focusing upon collagen-specific proline and hydroxyproline alongside precursors of hydroxyapatite including amorphous calcium phosphate (ACP) and carbonated apatite (CAP). Compared to cells cultured on uncoated substrates, spectral deconvolution analysis revealed a significant increase in collagen-specific proline and hydroxyproline by 4.01- and 5.57-fold, respectively, in the presence of PLL. This tied in with a 1.91-fold increase in immature ACP and a 9.32-fold reduction in mature CAP on PLL vs. controls (Figure 1). These unique low mineral signatures driven by PLL were associated with reduced alkaline phosphatase enzymatic activity and Alizarin Red staining in differentiation promoting conditions.

Conclusively, the combination of results from our own study and Buo et al. (2) suggest that improved survival of primary OBs on PLL may contribute to the enhanced transfection efficiency observed whilst concomitantly preventing further maturation. Similar studies utilizing alternative poly-cationic coatings such as polybrene (5, 6), DEAE-dextran (7), and protamine sulfate (8, 9) have reported comparable improved uptake of retroviruses, adenoviruses and lentiviruses. However, due to the considerable lack of studies performed on OBs or related-cell types, it is not clear if these coatings exert the same detriment to OB maturation or promote the maintenance of a proliferating immature state. The unknown effects of OBs cultured on various coatings and the implications on osteogenic development should therefore be thoroughly characterized ahead of use, using techniques such as Raman spectroscopy and mineral staining. In comparison, a variety of synthetic or purified ECM-derivatives including collagen, fibronectin and vitronectin among others (10–14) have been reported to induce osteogenesis *in vitro*. Whilst it is unknown what transduction efficiencies can be attained on these surfaces, the unrestricted progression from proliferative to differentiative phases should be considered as a potential alternative platform on which viral-mediated transduction can be performed on in the future.

## Conclusion

Together our experimental results suggest that although the use of PLL coatings may provide a cost-effective means to improve transfection efficiency, PLL could exert divergent unexpected effects on OB commitment and maturation (Figure 1). Important experimental differences between the two studies described here include PLL concentration and age of mice used for OB extraction. Nevertheless, given the broad range and sources of primary OBs that are used in our global bone research community, the importance in thoroughly investigating effects of PLL on OB commitment ahead of *in vitro* genetic manipulation is critical.

Paradoxically, PLL coatings appear to provide a fascinating opportunity to artificially restrict OB differentiation and mineralization in favor of unmineralized and pro-angiogenic matrix production (3) and thus could allow recapitulation of early developmental processes of bone growth and expansion. This approach could allow us to better define the regulators underlying early matrix production and decipher the mechanisms controlling matrix mineralization onset.

## Author Contributions

CC, AS, and AG conceived and drafted the paper. All authors contributed to the article and approved the submitted version.

## Conflict of Interest

The authors declare that the research was conducted in the absence of any commercial or financial relationships that could be construed as a potential conflict of interest.
